# Slab thickness tuning approach for solid-state strong coupling between photonic crystal slab nanocavity and a quantum dot

**DOI:** 10.1186/1556-276X-8-187

**Published:** 2013-04-23

**Authors:** Gengyan Chen, Jing-Feng Liu, Haoxiang Jiang, Xiao-Lu Zhuo, Yi-Cong Yu, Chongjun Jin, Xue-Hua Wang

**Affiliations:** 1 State Key Laboratory of Optoelectronic Materials and Technologies, School of Physics and Engineering, Sun Yat-sen University, Guangzhou 510275, China

**Keywords:** Photonic crystal slab nanocavity, Slab thickness, Quality factor, Mode volume, Strong coupling interaction, Cavity quantum electrodynamics

## Abstract

The quality factor and mode volume of a nanocavity play pivotal roles in realizing the strong coupling interaction between the nanocavity mode and a quantum dot. We present an extremely simple method to obtain the mode volume and investigate the effect of the slab thickness on the quality factor and mode volume of photonic crystal slab nanocavities. We reveal that the mode volume is approximatively proportional to the slab thickness. As compared with the previous structure finely optimized by introducing displacement of the air holes, via tuning the slab thickness, the quality factor can be enhanced by about 22%, and the ratio between the coupling coefficient and the nanocavity decay rate can be enhanced by about 13%. This can remarkably enhance the capability of the photonic crystal slab nanocavity for realizing the strong coupling interaction. The slab thickness tuning approach is feasible and significant for the experimental fabrication of the solid-state nanocavities.

## Background

Photonic crystals (PCs) [1-3] are artificial dielectric nanostructures with a periodic variation of dielectric function in the length scale of optical wavelength, and provide a unique way to control the decay kinetic of the quantum emitters inside the PCs due to photonic bandgaps and a strong inhomogeneity of electromagnetic fields [4-10]. Since many sophisticated and mature fabrication technologies developed in micro-electronics and opto-electronics can be applied to its fabrication, the PC slab, which is a thin semiconductor slab with two-dimensional (2D) periodicity along the slab plane, has been investigated energetically in depth both theoretically and experimentally [11-15]. Owing to the strong vertical optical confinement and the 2D photonic bandgap effect, the overall spontaneous emission rate of the quantum emitter inside the PC slab decreases substantially [14].

By introducing an artificial point defect into the PC slab, the PC slab nanocavity [3] can be formed. The point defect traps a localized nanocavity mode, which decays in inverse proportion to the quality factor of the PC slab nanocavity. The PC slab nanocavity and a single two-level quantum dot can realize the strong coupling interaction and thus constitute the solid-state strong coupling system (SSSCS) [16]. In this SSSCS, there is reversible exchange of a single photon between the quantum dot and the nanocavity mode before the photon leaks out of the nanocavity. The SSSCS realizes many fascinating but genuine quantum behaviors in cavity quantum electrodynamics [17], e.g., vacuum Rabi splitting [16,18,19] and lasing under strong coupling [20]. The SSSCS not only provides test beds for fundamental quantum physics but also has important applications in quantum information processing [21-23].

The realization of the strong coupling interaction relies on the condition that the coupling coefficient between the nanocavity mode and the quantum dot exceeds the intrinsic decay rate of the nanocavity [17]. To fulfill this condition, a great deal of efforts [24-27] have been devoted to design the nanocavities with the ultrahigh quality factor and ultrasmall mode volume.

To enhance the quality factor, various types of the PC slab nanocavities have been presented. The prominent types of the PC slab nanocavities with ultrahigh quality factor include the PC L3 nanocavity [25] and PC heterostructure nanocavity [27]. The PC L3 nanocavity is formed by missing three air holes in a line and displacing several pairs of air holes at both edges of the nanocavity, which can increase the quality factor substantially by following the principle that light should be confined gently in order to be confined strongly [25,26]. The PC heterostructure nanocavity is formed by adjusting the lattice constant of several rows of air holes and introducing mode gap difference in the PC slab waveguide, which can obtain unprecedentedly ultrahigh quality factor by following the same principle [27]. Obviously, this principle requires the elaborately designed and optimized PC slab nanocavity with highly fine tuning of the positions and radii of the air holes around the nanocavity center, commonly up to the nanometer scale accuracy, which is a great challenge due to the technical limits of the semiconductor process [28]. However, the effect of the PC slab thickness on the quality factor has not been reported.

Besides the quality factor, another important parameter for the realization of the strong coupling interaction is the mode volume of the nanocavity. Traditionally, the mode volume is calculated by simulating and then integrating the electric field distribution of the nanocavity mode around the whole nanocavity region [24-26,29] (see Equation 6). This is a rather time-consuming and difficult task. Obviously, a simple and efficient numerical method for the calculation of mode volume is desirable and remains a challenge so far.

In this paper, we present an extremely simple method to determine the volume of a nanocavity mode and investigate the effect of the slab thickness on the quality factor and mode volume of the PC slab nanocavities based upon projected local density of states for photons [30]. It is found that the mode volume monotonously expands with the increasing slab thickness. As compared with the previous structure finely optimized by introducing displacement of the air holes, via tuning the slab thickness, the quality factor can be enhanced by about 22%, and the ratio between the coupling coefficient and the nanocavity decay rate can be enhanced by about 13%. Our work provides a feasible approach to manipulate the quality factor and mode volume in the experiment. This is significant for the realization of the strong coupling interaction between the PC slab nanocavity and a quantum dot, which has important applications in quantum information processing [21-23].

## Methods

The optical properties of an arbitrary dielectric nanostructure can be characterized by the projected local density of states (PLDOS) [30], which is defined as follows:

(1) $$\rho \left(r_{0},\omega ,\hat{d}\right)=\sum_{\lambda }{\left|\hat{d}\cdot E_{\lambda }\left(r_{0}\right)\right|}^{2}\delta \left(\omega -\omega_{\lambda }\right),$$

where **r**_0_ is the location; *ω*, the frequency; $\hat{d}$, the orientation; and **E**_*λ*_(**r**) and *ω*_*λ*_, the normalized eigen electric field and eigen frequency of the *λ*th eigenmode of the nanostructure, respectively.

In an ideal single-mode nanocavity without loss, the PLDOS can be expressed as follows:

(2) $$\rho_{c}\left(r_{0},\omega ,\hat{d}\right)={\left|\hat{d}\cdot E_{c}\left(r_{0}\right)\right|}^{2}\delta \left(\omega -\omega_{c}\right),$$

where **E**_*c*_(**r**) and *ω*_*c*_ are the normalized eigen electric field and eigen frequency of the nanocavity mode, respectively.

Considering the loss, the PLDOS of a realistic single-mode nanocavity can be generalized to Lorentz function [31] as follows:

(3) $$\rho_{c}\left(r_{0},\omega ,\hat{d}\right)=\frac{{\left|\hat{d}\cdot E_{c}\left(r_{0}\right)\right|}^{2}}{\pi }\frac{\kappa /2}{{\left(\omega -\omega_{c}\right)}^{2}+{\left(\kappa /2\right)}^{2}},$$

where *κ* = *ω*_*c*_ / *Q* is the decay rate of the realistic nanocavity with loss and *Q* represents the quality factor. Apparently, when *κ* is infinitely small, Equation 3 of the loss nanocavity approaches to Equation 2 of the lossless nanocavity.

For a specific location **r**_0_ and orientation $\hat{d}$, the PLDOS of the loss nanocavity reaches its peak value *ρ*_*cp *_at *ω *= *ω*_*c *_as follows:

(4) $$\rho_{\mathrm{cp}}=\frac{2{\left|\hat{d}\cdot E_{c}\left(r_{0}\right)\right|}^{2}}{\kappa \pi },$$

so we can further simplify the PLDOS of the loss nanocavity as follows:

(5) $$\rho_{c}\left(r_{0},\omega ,\hat{d}\right)=\frac{\kappa \rho_{\mathrm{cp}}}{2}\frac{\kappa /2}{{\left(\omega -\omega_{c}\right)}^{2}+{\left(\kappa /2\right)}^{2}}.$$

The mode volume of the nanocavity characterizes the confinement and localization of the nanocavity mode and is defined as follows [26]:

(6) $$V=\frac{\int \epsilon_{r}\left(r\right){\left|{\tilde{E}}_{c}\left(r\right)\right|}^{2}d^{3}r}{\text{max}\left[\epsilon_{r}\left(r\right){\left|{\tilde{E}}_{c}\left(r\right)\right|}^{2}\right]},$$

where *ϵ*_*r*_(**r**) is the relative dielectric constant and ${\tilde{E}}_{c}\left(r\right)$ is the electric field of the nanocavity mode. The numerator is the normalization factor of the nanocavity mode field. The calculation of the normalization factor is rather difficult and time-consuming. However, since we can directly use the normalized nanocavity mode field **E**_*c*_(**r**) adopted in Equations 2 to 4, we do not need to calculate this normalization factor. With the normalized nanocavity mode field **E**_*c*_(**r**), Equation 6 can be simplified as follows:

(7) $$V=\frac{1}{\text{max}\left[\epsilon_{r}\left(r\right){\left|E_{c}\left(r\right)\right|}^{2}\right]}.$$

**Figure 1:**
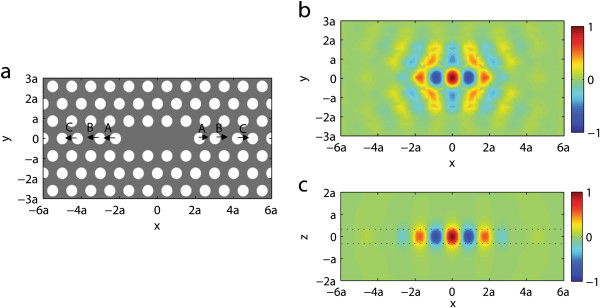
**The structure diagram and nanocavity mode of the PC L3 nanocavity. **(**a**) Cross section on the central plane (*z *= 0 plane) of the PC L3 nanocavity. Gray region is the dielectric slab, and white regions are the air holes. *A*, *B*, and *C* denote the displacements of the first, second, and third nearest pair of air holes, respectively. The air holes are moved outward along the *x *direction, denoted by the arrows. (**b**, **c**) *E*_*y *_component of the electric field **E**_*c*_(**r**) of the PC L3 nanocavity mode with the air hole displacements *A* = 0.2*a*, *B *= 0.025*a*, and *C *= 0.2*a *(b) on *z* = 0 plane and (c) on *y* = 0 plane, respectively. The electric field distribution is normalized by the electric field maximum at the center of the nanocavity **r**_0*m *_= (0, 0, 0). The two dotted lines denote the top and bottom surfaces of the slab.

We assume that *ϵ*_*r*_(**r**)|**E**_*c*_(**r**)|^2^ reaches to its maximum at location **r**_0*m*_ and denote the direction of the vector **E**_*c*_(**r**_0*m*_) at this location as ${\hat{d}}_{m}$. For most of the PC slab nanocavities, **r**_0*m*_ and ${\hat{d}}_{m}$ are known before the simulation. For instance, for the PC L3 nanocavity, **r**_0*m*_ is at the nanocavity center and ${\hat{d}}_{m}$ is perpendicular to the line of centers of the three defect air holes, as will be shown in Figure 1b.

By substituting Equation 4 with Equation 7, we can obtain the following:

(8) $$V=\frac{2}{\pi \kappa \rho_{\mathrm{cpm}}\epsilon_{r}\left(r_{0m}\right)},$$

where $\rho_{\mathrm{cpm}}=\rho_{c}\left(r_{0m},\omega_{c},{\hat{d}}_{m}\right)$ is the peak value of the PLDOS at the location **r**_0*m*_ along the direction ${\hat{d}}_{m}$. Therefore, as soon as the PLDOS at the location **r**_0*m*_ along the direction ${\hat{d}}_{m}$ is calculated by various numerical methods, *ω*_*c*_, *κ*, and *ρ*_*cpm*_ can be determined by fitting the PLDOS by the Lorentz function of Equation 5. Based on them, we can finally obtain the mode volume of the nanocavity by Equation 8 and the quality factor of the nanocavity by *Q* = *ω*_*c*_ / *κ*.

Traditionally [24-26,29], the mode volume of the PC slab nanocavity is calculated directly by Equation 6. By this method, the electric field distribution of the nanocavity mode around the whole nanocavity region needs to be simulated and then integrated. This is rather time-consuming. In contrast, using our method of Equation 8, we can calculate the mode volume simply and efficiently. We just need to calculate the PLDOS at only one known location and along one known direction, which make the calculation of the mode volume very efficient.

As mentioned previously, the realization of the strong coupling interaction requires that the coupling coefficient *g* exceeds the intrinsic decay rate of the nanocavity mode *κ*. Thus, the most important issue for the realization of the strong coupling is to increase the ratio of $g/\kappa \propto Q/\sqrt{V\omega_{c}}$[16], which can be obtained using our method.

## Results and discussion

To compare our slab thickness tuning approach with previous air hole displacement approach, we investigate the PC L3 nanocavity that was finely optimized by the air hole displacement approach in [26], as shown in Figure 1a.

The 2D PC slab is composed of silicon (refractive index *n* = 3.4) with a triangular lattice of air holes. The lattice constant is *a* = 420 nm. The slab thickness is *d* = 0.6*a*, and the air hole radius is *r* = 0.29*a*. The PC L3 nanocavity is formed by missing three air holes in a line in the center of the PC slab and can be further optimized by firstly tuning the displacement *A* of the first nearest pair of air holes and then tuning the displacement *B* of the second nearest pair of air holes and, finally, the displacement *C* of the third nearest pair of air holes, as shown in Figure 1a.

The *E*_*y*_ component of the electric field **E**_*c*_(**r**) of the nanocavity mode is shown in Figure 1b,c, obtained by finite-difference time-domain method [32]. This spatial distribution is typical among all the PC L3 nanocavities. Obviously, most electromagnetic energy of the nanocavity mode is localized in the three missed air holes due to the 2D photonic bandgap effect and is also confined inside the slab by the total internal reflection. The *E*_*y*_ component reaches its maximum at the nanocavity center **r**_0*m*_ = (0, 0, 0).

**Figure 2:**
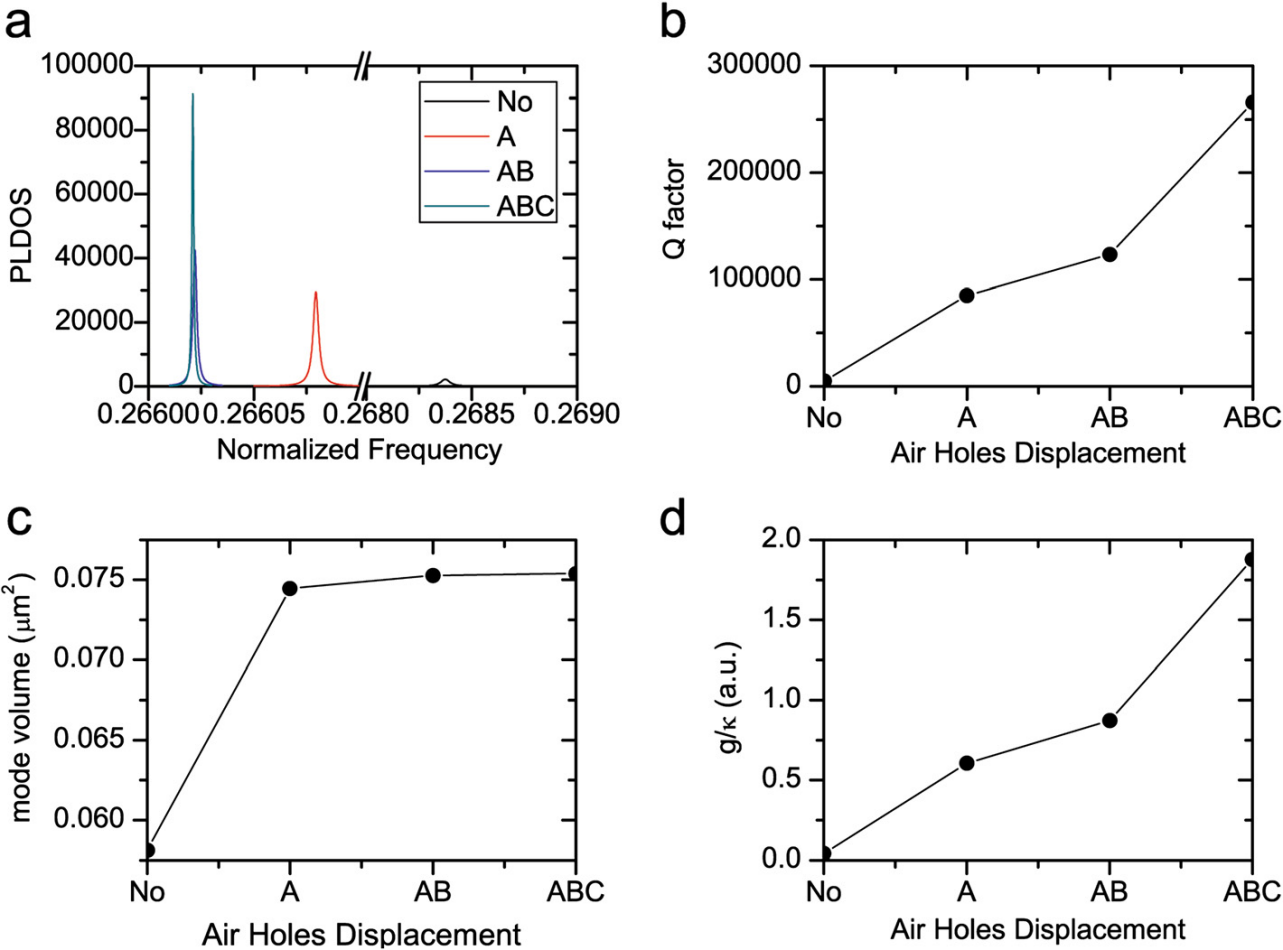
**The PC L3 nanocavities with the slab thickness *d *= 0.6*a *and different air hole displacements. **Including ‘no displacement’ (denoted as No), ‘*A *= 0.2*a*’ (denoted as A), ‘*A* = 0.2*a*, *B *= 0.025*a*’ (denoted as AB), and ‘*A *= 0.2*a*, *B *= 0.025*a*, *C *= 0.2*a*’ (denoted as ABC). (**a**) The PLDOS at the center of the PC L3 nanocavities, orientating along the *y* direction, normalized by the PLDOS in vacuum as *ω*^2 ^/ 3*π*^2^*c*^3^. (**b**) The quality factor. (**c**) The mode volume. (**d**) The ratio of *g*/*κ*.

First of all, we focus on the cases where the slab thickness is fixed at *d* = 0.6*a*, and the air hole displacements *A*, *B*, and *C* are tuned and optimized in turn according to [26]. The PLDOS of the non-optimized and the three optimized PC L3 nanocavities are calculated, and the results are shown in Figure 2a. Obviously, as the PC L3 nanocavity is further tuned and optimized, we find that (a) the resonant frequency slightly shifts to the lower frequency, and (b) the decay rate of the PC L3 nanocavity, i.e., the full-width at half maximum of Lorentz function of the PLDOS, is further suppressed, which leads to the remarkable increase of quality factor, as shown in Figure 2b.

However, as the three pairs of air holes near the PC L3 nanocavity center are further moved outward, the nanocavity mode is confined inside the nanocavity more and more gently [25], as shown in Figure 1b. Consequently, the mode volume of nanocavity mode becomes large, as shown in Figure 2c. The calculated mode volume of the optimized PC L3 nanocavity with air hole displacements *A* = 0.2*a*, *B* = 0.025*a*, and *C* = 0.2*a* is 0.0754 μm^3^, which agrees well with the reported mode volume as 0.074 μm^3^ in [26]. This excellent agreement validates our method of Equation 8 for calculating the mode volume.

Based on the calculated quality factor, resonant frequency, and mode volume, we can obtain the ratio of *g*/*κ*, which assesses the PC L3 nanocavity for the realization of the strong coupling interaction between a quantum dot and the nanocavity mode. As the air hole displacements *A*, *B*, and *C* are tuned and optimized in turn, *g*/*κ* is also enhanced remarkably, as shown in Figure 2d, which is mainly due to the sharply decreased decay rate *κ* of the nanocavity.

**Figure 3:**
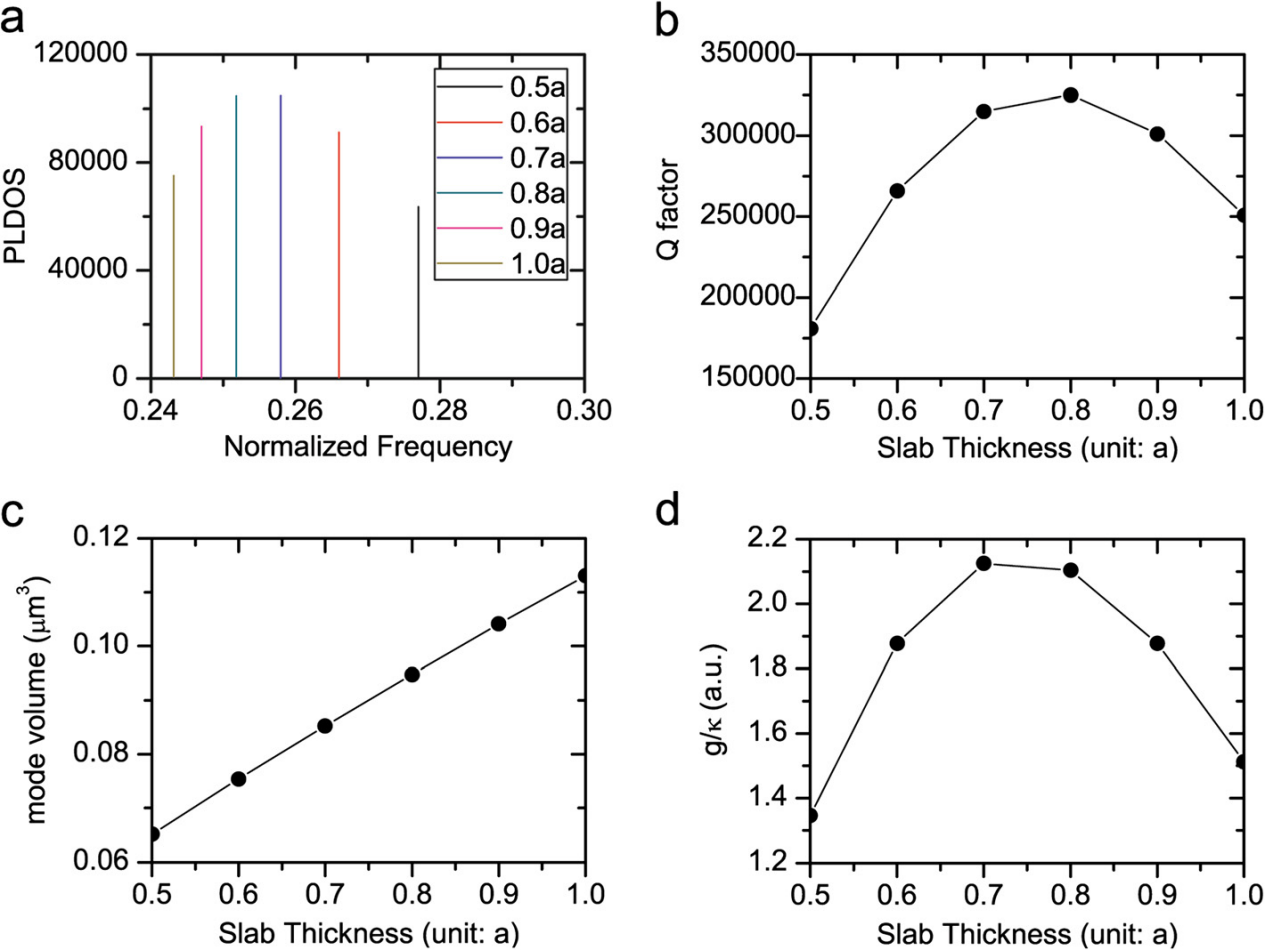
**The PC L3 nanocavities with different slab thicknesses. **The air hole displacements are *A *= 0.2*a*, *B *= 0.025*a*, and *C *= 0.2*a*. (**a**) The PLDOS at the center of the PC L3 nanocavities, orientating along the *y* direction, normalized by the PLDOS in vacuum as *ω*^2 ^/ 3*π*^2^*c*^3^. Each ‘vertical line’ is actually a Lorentz function with small full-width at half maximum. (**b**) The quality factor. (**c**) The mode volume. (**d**) The ratio of *g*/*κ*.

Actually, based on the previous optimized PC L3 nanocavity with air hole displacements *A* = 0.2*a*, *B* = 0.025*a*, and *C* = 0.2*a*, we can further enhance the quality factor by optimizing its slab thickness. We calculate the PLDOS of the PC L3 nanocavities with different slab thicknesses. The results are shown in Figure 3a. As the slab thickness increases from *d* = 0.5*a* to *d* = 1.0*a*, the resonant wavelength of the PC L3 nanocavity also increases, and hence, the resonant frequency decreases substantially.

As shown in Figure 3b, as we tune the slab thickness, the quality factor varies remarkably and reaches its maximum at the slab thickness *d* = 0.8*a*. By the slab thickness tuning approach, we can further optimize the quality factor from *Q* = 265,985 for *d* = 0.6*a* in [26] to *Q* = 325,121 for *d* = 0.8*a*, with increase of about 22%. This optimized PC L3 nanocavity with higher quality factor is desirable and beneficial to the realization of the SSSCS.

Along the vertical (*z*) direction perpendicular to the slab plane, the electric field of the nanocavity mode is mostly confined inside the slab by the total internal reflection, as shown in Figure 1c. Thus, when the slab thickness increases from *d* = 0.5*a* to *d* = 1.0*a*, the nanocavity mode is confined inside the slab more and more loosely, and hence, the mode volume expands almost linearly along with the increasing slab thickness, as shown in Figure 3c.

As we tune the slab thickness, the ratio of *g*/*κ* varies substantially and also reaches its maximum at the slab thickness *d* = 0.7*a*. The optimized *g*/*κ* at the slab thickness *d* = 0.7*a* is about 13% higher than that of *d* = 0.6*a* in [26]. From Figure 3d, we can notice that there is an optimization region for the slab thickness from *d* = 0.7*a* to 0.8*a*, in which the ratio *g*/*κ* varies little. This is very beneficial for the experimental fabrication of the PC L3 nanocavity.

In a word, the nanocavity mode is confined inside the PC L3 nanocavity by the 2D photonic bandgap effect along the slab plane and also by the total internal reflection in the out-of-plane direction. Thus, as we displace the air holes near the nanocavity center outwards or as we increase the slab thickness, the nanocavity mode is confined inside the nanocavity more gently and loosely. In this case, the mode volume of the nanocavity mode expands, and the electric field maximum at the nanocavity center decreases, which results in the decrease of the coupling coefficient *g* between a quantum dot and the nanocavity mode. Since the ratio *g*/*κ* between the coupling coefficient and the nanocavity decay rate characterizes the capability of the PC L3 nanocavity for realizing the strong coupling interaction between a quantum dot and the nanocavity mode, we should pay more attention to enhance the ratio *g*/*κ*, instead of only pursuing higher quality factor.

## Conclusions

In summary, we have presented a simple and efficient method based upon the projected local density of states for photons to obtain the mode volume of a nanocavity. The effect of the slab thickness on the quality factor and mode volume of photonic crystal slab nanocavities has been investigated, which both play pivotal roles in cavity quantum electrodynamics.

We find that the mode volume is approximatively proportional to the slab thickness. Furthermore, by tuning the slab thickness, the quality factor can be increased by about 22%, and the ratio *g*/*κ* between the coupling coefficient and the nanocavity decay rate can be enhanced by about 13%, as compared with the previous PC L3 nanocavity that is finely optimized by introducing displacement of the air holes at both edges of the nanocavity. Based on these results, we can conclude that the optimization of the slab thickness can remarkably enhance the capability of the PC slab nanocavity for the realization of the strong coupling interaction between a quantum dot and the nanocavity mode. The slab thickness tuning approach is feasible and significant for the experimental fabrication of the solid-state nanocavities.

## Abbreviations

PC: photonic crystal; PLDOS: projected local density of states; SSSCS: solid-state strong coupling system; 2D: two-dimensional.

## Competing interests

The authors declare that they have no competing interests.

## Authors’ contributions

GC proposed the method for the mode volume, performed the numerical simulations, interpreted the simulation results, and drafted the manuscript. J-FL anticipated the derivation of equations and the interpretation of numerical results. HJ anticipated the coding of the numerical programs. X-LZ and Y-CY anticipated the numerical simulations and the interpretation of numerical results. CJ and X-HW conceived the study, proposed the slab thickness tuning approach, and revised the manuscript substantially. All authors read and approved the final manuscript.

## Authors’ information

GC, X-LZ, and Y-CY are Ph.D. students in Sun Yat-sen University. J-FL and HJ are Ph.D. degree holders in Sun Yat-sen University. CJ and X-HW are professors of Sun Yat-sen University.
